# SOD2 Activity Is not Impacted by Hyperoxia in Murine Neonatal Pulmonary Artery Smooth Muscle Cells and Mice

**DOI:** 10.3390/ijms16036373

**Published:** 2015-03-19

**Authors:** Anita Gupta, Marta Perez, Keng Jin Lee, Joann M. Taylor, Kathryn N. Farrow

**Affiliations:** Department of Pediatrics, Northwestern University Feinberg School of Medicine, 310 E. Superior St., Chicago, IL 60611, USA; E-Mails: agupta@luriechildrens.org (A.G.); mtperez@luriechildrens.org (M.P.); kengjin.lee@northwestern.edu (K.J.L.); j-hinz@northwestern.edu (J.M.T.)

**Keywords:** bronchopulmonary dysplasia, superoxide dismutase 2 (SOD2), right ventricular hypertrophy, pulmonary hypertension

## Abstract

Pulmonary hypertension (PH) complicates bronchopulmonary dysplasia (BPD) in 25% of infants. Superoxide dismutase 2 (SOD2) is an endogenous mitochondrial antioxidant, and overexpression protects against acute lung injury in adult mice. Little is known about SOD2 in neonatal lung disease and PH. C57Bl/6 mice and isogenic SOD2+/+ and SOD2−/+ mice were placed in room air (control) or 75% O_2_ (chronic hyperoxia, CH) for 14 days. Right ventricular hypertrophy (RVH) was assessed by Fulton’s index. Medial wall thickness (MWT) and alveolar area were assessed on formalin fixed lung sections. Pulmonary artery smooth muscle cells (PASMC) were placed in 21% or 95% O_2_ for 24 h. Lung and PASMC protein were analyzed for SOD2 expression and activity. Oxidative stress was measured with a mitochondrially-targeted sensor, mitoRoGFP. CH lungs have increased SOD2 expression, but unchanged activity. SOD2−/+ PASMC have decreased expression and activity at baseline, but increased SOD2 expression in hyperoxia. Hyperoxia increased mitochondrial ROS in SOD2+/+ and SOD2−/+ PASMC. SOD2+/+ and SOD2−/+ CH pups induced SOD2 expression, but not activity, and developed equivalent increases in RVH, MWT, and alveolar area. Since SOD2−/+ mice develop equivalent disease, this suggests other antioxidant systems may compensate for partial SOD2 expression and activity in the neonatal period during hyperoxia-induced oxidative stress.

## 1. Introduction

Bronchopulmonary dysplasia (BPD), a common complication of preterm birth, is associated with pulmonary hypertension (PH) in approximately 25% of infants with moderate to severe BPD. These infants are significantly at risk for prolonged hospital stays, increased morbidity including need for tracheostomy, and increased mortality. Despite the high risks for poor outcomes in these patients, there is no evidence-based therapy for this condition, and new treatment options are urgently needed [1,2,3,4,5,6].

The cGMP pathway is known to be important in regulating neonatal pulmonary vascular tone. We recently reported that hyperoxia exposure initially increases reactive oxygen species (ROS) in the mitochondrial matrix followed by the cytoplasm of ovine fetal pulmonary artery smooth muscle cells. This increased oxidative stress leads to increased phosphodiesterase-5 (PDE5) activity and decreased cGMP levels in response to exogenous NO [7,8,9]. Furthermore, we recently utilized a hyperoxic mouse model of neonatal lung injury to demonstrate that chronic hyperoxia (CH) increased PDE5 activity and decreased cGMP in the small pulmonary arteries (PA) *in vivo*. Treating the mice with sildenafil during hyperoxia effectively blocked PDE5 activity, pulmonary vascular remodeling, and development of right ventricular hypertrophy, solidifying the role for hyperoxia-induced PDE5 activity in PH and right ventricular hypertrophy (RVH) in this model [10].

Since oxidant-induced disruption of cGMP signaling appears to be critical in the disease pathogenesis of BPD-associated PH, we sought to determine if antioxidant enzymes would play a protective role in oxygen-induced lung injury. The major antioxidant enzymes found in eukaryotes are superoxide dismutase (SOD), glutathione peroxidase, and catalase. Three different SOD isoforms are found in mammals, and all SODs catalyze the conversion of superoxide anions to hydrogen peroxide (H_2_O_2_). Cytosolic copper/zinc SOD (Cu/ZnSOD or SOD1) is the predominant SOD in most cells and tissues (70%–80% of total cellular SOD activity). Extracellular SOD (EC-SOD or SOD3) represents only a small amount of total cellular SOD activity, but it is highly expressed in the matrix-rich wall of arteries [11]. Overexpression of SOD3 has been shown previously by other investigators to be beneficial in animal models of hyperoxia-induced lung injury [12]. Furthermore, recombinant human superoxide dismutase (rhSOD), which is Cu/ZnSOD, has been trialed in human infants to prevent BPD, and while rhSOD given intratracheally was safe, it did not prevent BPD or death [13].

Since our previous studies implicated mitochondrial oxidative stress [7,8], we hypothesized that manganese SOD (MnSOD) or SOD2 might be a more effective therapeutic target than SOD1. SOD2 is located in the mitochondrial matrix and contributes 10%–20% of the total SOD activity in the cell. The presence of SOD2 at the matrix side on the inner mitochondrial membrane allows it to play a critical role in protecting the mitochondria from oxidant stress by enzymatically scavenging superoxide anions that are produced as a byproduct of the electron transport chain [14]. Previous animal studies have shown SOD2 overexpression is protective against acute lung injury in adult mice [15]. Conversely, heterozygous SOD2−/+ adult mice have increased susceptibility to myocardial injury with aging [16]. Previous studies in adult SOD2−/+ mice have suggested no difference in death rates with severe hyperoxia exposure for 48 h (85%–100% O_2_) in SOD2 heterozygous mice *vs.* isogenic wild-type mice [17]. However, virtually nothing is known about the interaction of SOD2 and cGMP signaling, nor about the role of SOD2 in PH in neonatal animal models of hyperoxic lung injury.

In the present study, we used a previously described murine model of hyperoxic lung injury to approximate BPD and BPD-associated PH [18,19]. Rodents are well suited for the study of BPD because their lungs at birth are structurally similar to human neonates born at 24 to 28 weeks of gestation [10,20]. Neonatal mice exposed to hyperoxia for 14 days develop alveolar simplification, decreased vessel density, vascular remodeling, and RVH, similar to what is seen in infants with BPD-associated PH [10]. Here, we determine the impact of hyperoxia on SOD2 expression and activity in wild-type C57Bl6 mice. Furthermore, we investigated in primary isolated pulmonary artery smooth muscle cells (PASMC) whether hyperoxia impacts SOD2 expression or activity and mitochondrial matrix oxidative stress. Lastly, we examined whether SOD2 plays a critical role in pulmonary vascular disease by exposing SOD2 heterozygous mice to our mouse model of hyperoxia-induced PH.

## 2. Results and Discussion

### 2.1. CH Exposed Lungs Have Increased SOD2 Expression but not Increased SOD2 Activity

**Figure 1:**
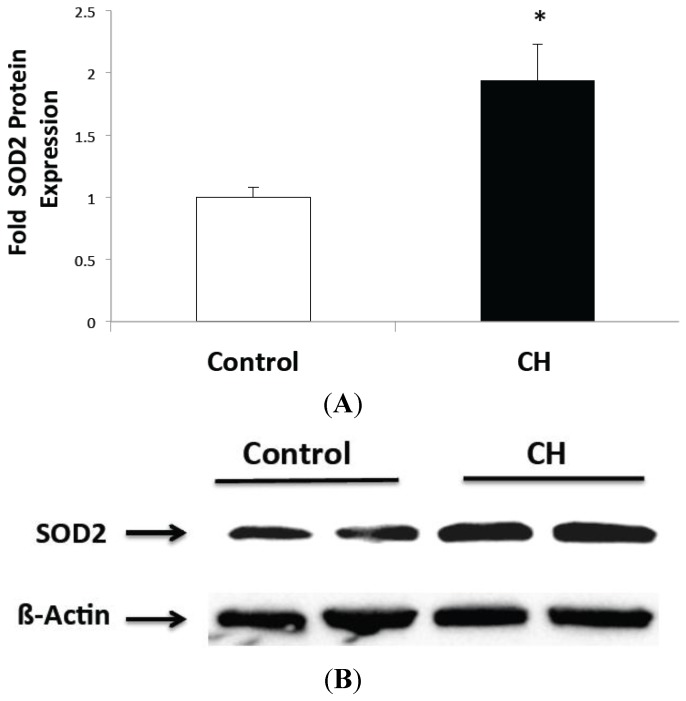

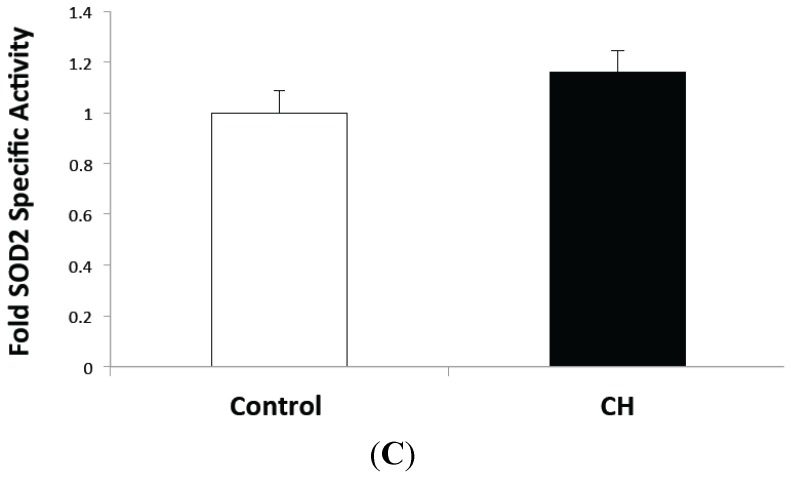
Chronic hyperoxia (CH) exposure increased SOD2 protein expression but not activity in mouse lungs. (**A**) Expression was measured in lung protein by Western blot in control and CH mice (*n* = 12 per group, * *p* < 0.05 *vs.* control); (**B**) Representative Western blot of SOD2 expression is shown with corresponding β-actin; and (**C**) SOD2 enzymatic activity was measured with a commercially available activity assay in control and CH mice (*n* = 20 per group). Data are shown as fold mean ± SEM.

SOD2 protein levels were 1.9-fold higher in the CH-exposed whole lung compared with room air controls (*p* < 0.05, Figure 1A). Representative Western blot images are shown in Figure 1B. However, SOD2 enzymatic activity did not change in CH-exposed whole lung compared with controls (Figure 1C).

### 2.2. Hyperoxia Increased SOD2 Protein Expression in both SOD2+/+ and SOD2−/+ PASMC but not SOD2 Activity

**Figure 2:**
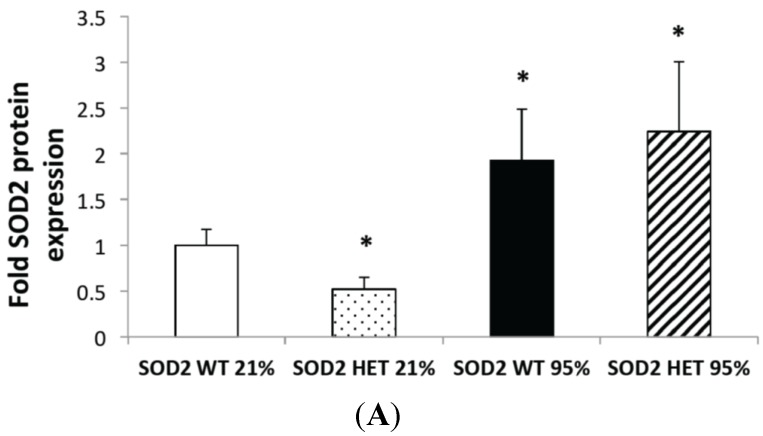

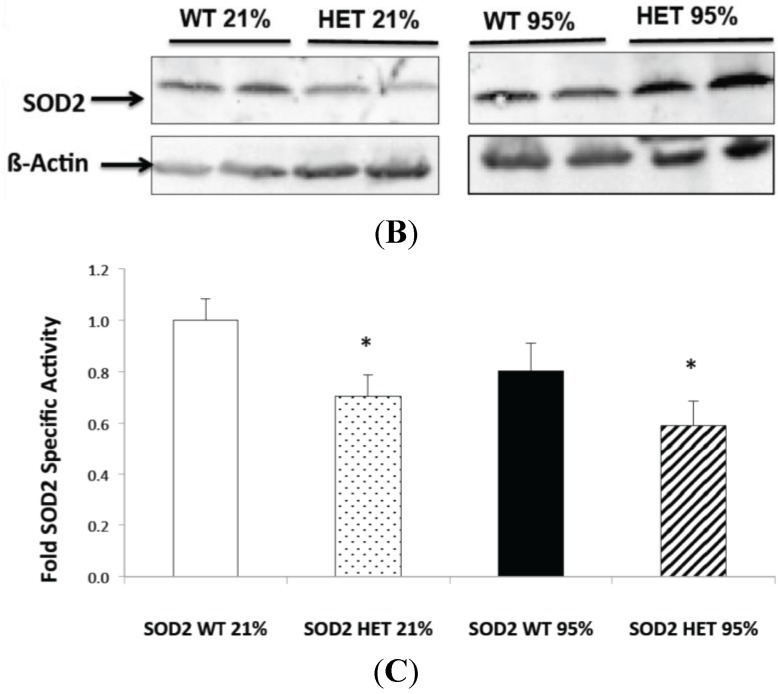
Hyperoxia increased SOD2 protein expression but not activity in pulmonary artery smooth muscle cells (PASMC) from SOD2−/+ and SOD2+/+ mice. (**A**) PASMC were isolated from both SOD2−/+ (HET) and SOD2+/+ (wild-type, WT) mice. PASMC were exposed to hyperoxia (95% O_2_) for 24 h. SOD2 expression was measured by Western blot in lysates from WT and HET PASMC (*n* = 6 for WT PASMC and *n* = 10 for HET PASMC) in both room air and hyperoxia (* *p* < 0.05 *vs.* SOD2 WT in 21% O_2_). Data are shown as mean ± SEM; (**B**) Representative Western blots of SOD2 expression are shown with corresponding β-actin; and (**C**) PASMC from HET and WT mice were assayed for SOD2 activity using a commercially available activity assay (*n* = 14 for WT PASMC in 21% O_2_ and *n* = 12 for all other groups; * *p* < 0.05 *vs.* SOD2 WT in 21% O_2_). Data are shown as mean ± SEM.

PASMC were isolated from SOD2−/+ and SOD2+/+ mice and exposed to 95% O_2_ for 24 h to determine the direct effect of hyperoxia on SOD2 in the pulmonary vascular smooth muscle. As expected, SOD2−/+ PASMC had decreased SOD2 protein expression (0.43 ± 0.1-fold, *p* < 0.05) and activity (0.7 ± 0.08-fold, *p* < 0.05) at baseline relative to isogenic SOD2+/+ controls (Figure 2A,C). SOD2 protein expression was two-fold higher in the hyperoxia-exposed PASMC from SOD2−/+ and SOD2+/+ controls (*p* < 0.05, Figure 2A). Representative Western blots are shown in Figure 2B. However, SOD2 activity was not statistically significantly different in either hyperoxia-exposed SOD2−/+ or SOD2+/+ PASMC *vs.* matched room air controls (Figure 2C).

### 2.3. Mitochondrial Matrix Oxidative Stress Is Increased at Baseline and after Hyperoxia in SOD2−/+ PASMC Relative to Wild-Type Controls

**Figure 3:**
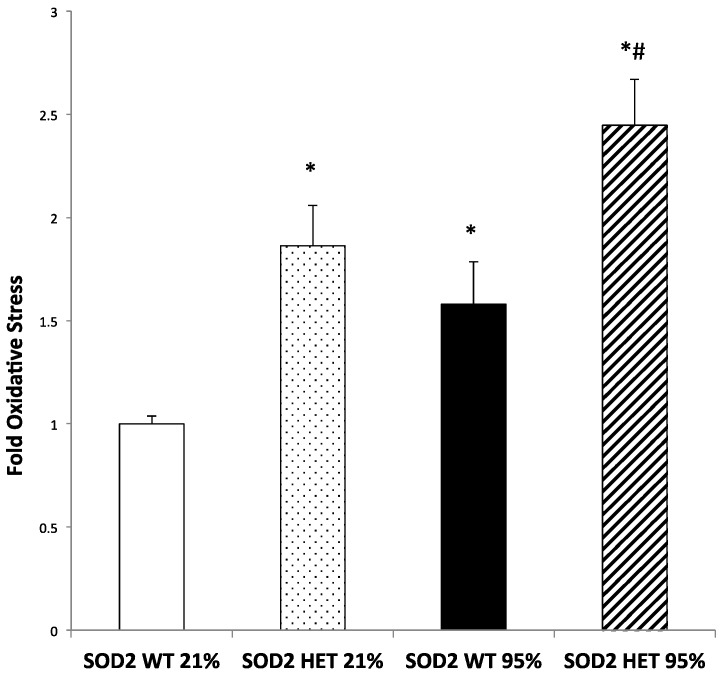
Hyperoxia increased ROS in both SOD2+/+ and SOD2−/+ PASMCs. PASMC were isolated from both SOD2−/+ (HET) and SOD2+/+ (WT) mice. PASMC were exposed to hyperoxia (95% O_2_) for 24 h. We utilized a targeted ratiometric redox-sensitive GFP probe (mitoRoGFP) to measure oxidative stress. After obtaining ratiometric measurements of RoGFP using dual laser flow cytometry, the sensor was calibrated by maximally reducing it with dithiothreitol (DTT, 1 mM) and maximally oxidizing it with t-butyl hydroperoxide (TBH, 1 mM). Data are shown as mean fold oxidative stress relative to SOD2+/+ (WT) PASMC in 21% O_2_ ± SEM (*n* = 7 (WT 21% O_2_), 9 (HET 21% O_2_), 8 (WT 95% O_2_), and 10 (HET 95% O_2_); * *p* < 0.05 *vs.* SOD2 WT in 21% O_2_, # *p* < 0.05 *vs.* SOD2 HET in 21% O_2_).

We have previously demonstrated that exposure to hyperoxia increases mitochondrial matrix oxidative stress followed by an increase in cytosolic oxidative stress [8]. To evaluate if oxidative stress is increased in the PASMCs isolated from SOD2−/+ PASMC, we used the previously characterized ratiometric thiol protein sensor, mitoRoGFP. PASMCs from SOD2−/+ mice were found to have increased oxidative stress in the mitochondrial matrix at baseline as compared to PASMCs from SOD2+/+ mice (*p* < 0.05, Figure 3). After exposure to 24 h of hyperoxia, SOD2−/+ PASMC had increased oxidative stress compared to room air controls, and the SOD2−/+ PASMC had an exaggerated response to hyperoxia as compared to SOD2+/+ PASMCs (*p* < 0.05, Figure 3).

### 2.4. SOD2−/+ PASMC Have Increased PDE5 Activity at Baseline

**Figure 4:**
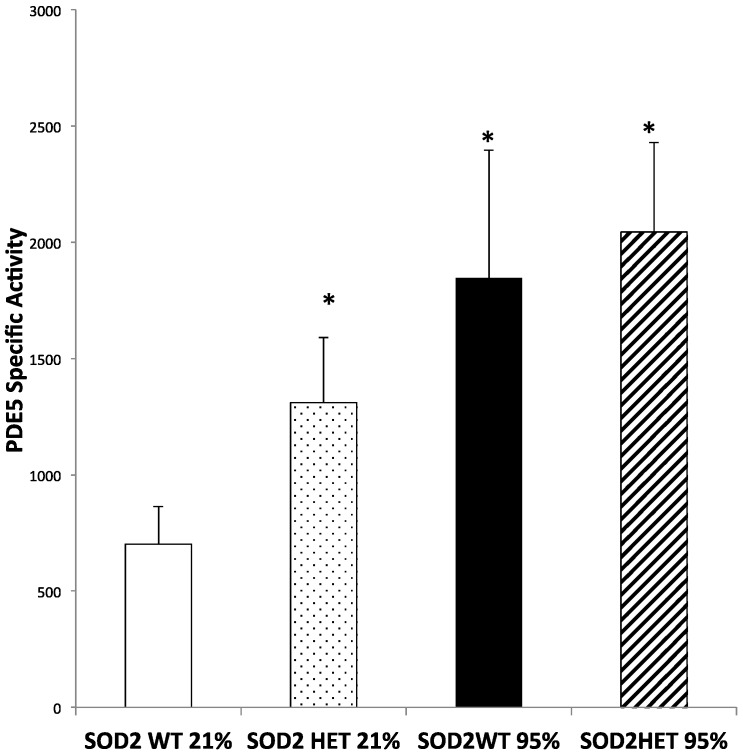
SOD2−/+ PASMC have increased phosphodiesterase 5 (PDE5) activity at baseline. PASMC were isolated from both SOD2−/+ (HET) and SOD2+/+ (WT) mice. PASMC were exposed to hyperoxia (95% O_2_) for 24 h. PDE5 activity was measured in PASMC lysates using a commercially available assay, and results are reported as pmol cGMP hydrolyzed/mg/minute (*n* = 7 (WT 21% O_2_), 5 (HET 21% O_2_), 4 (WT 95% O_2_), 7 (HET 95% O_2_); * *p* < 0.05 *vs.* WT 21% O_2_). Data are shown as mean ± SEM.

We have previously demonstrated that increased mitochondrial matrix oxidative stress is associated with increased PDE5 activity in ovine PASMC [7,8]. Therefore, we hypothesized that the increased mitochondrial matrix oxidative stress observed in the SOD2−/+ PASMC (Figure 3) would lead to increased PDE5 activity. In PASMCs in room air, SOD2−/+ PASMC had significantly increased PDE5 activity compared to SOD2+/+ PASMC (*p* < 0.05, Figure 4). Consistent with our previous results [8], hyperoxia exposure led to increased PDE5 activity in SOD2+/+ PASMC *vs.* those in room air (*p* < 0.05, Figure 4). However, there was no difference in the PDE5 activity in PASMC in 95% O_2_ between SOD2−/+ and SOD2+/+ PASMC (Figure 4).

### 2.5. SOD2−/+ Mice Develop Equivalent Lung and Vascular Disease in Chronic Hyperoxia (CH) Relative to SOD2+/+ Littermates

**Figure 5:**
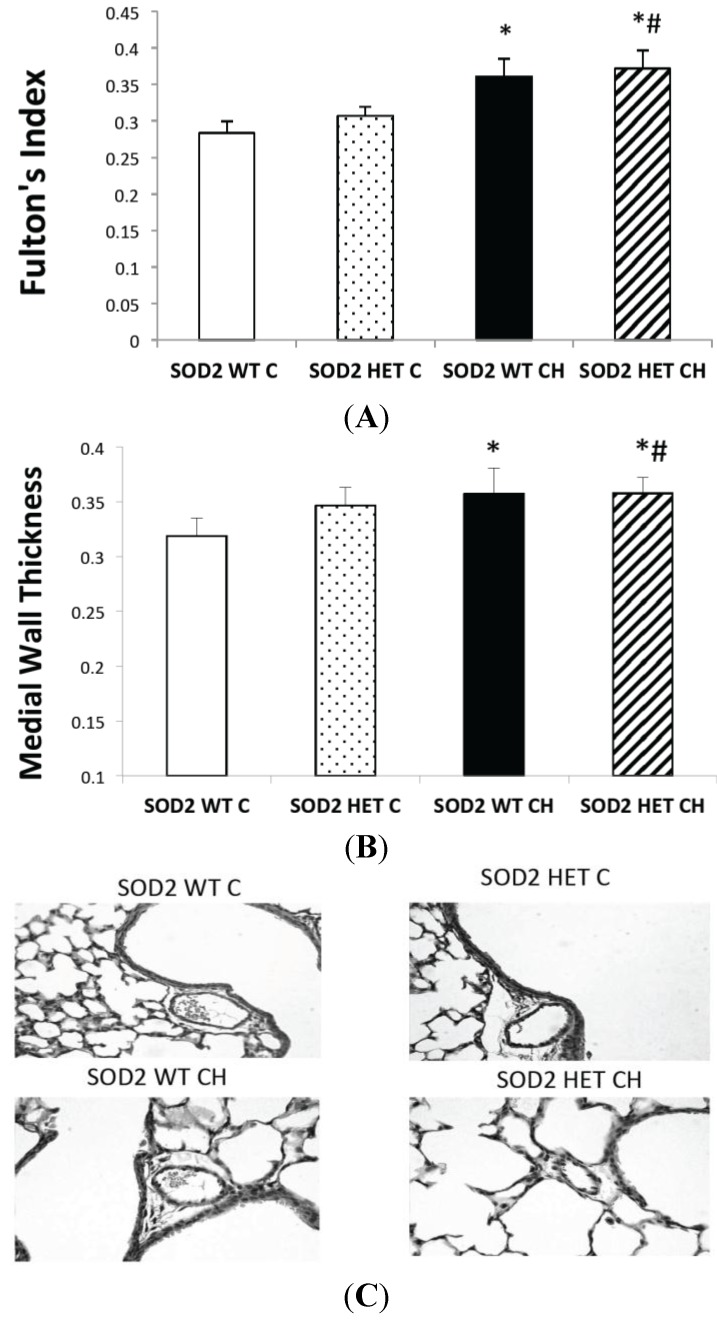
SOD2−/+ mice develop equivalent pulmonary vascular disease in chronic hyperoxia (CH) relative to SOD2+/+ littermates. Neonatal mice (SOD2−/+ (HET) and isogenic SOD2+/+ (WT) littermates) were exposed to chronic hyperoxia (CH) or room air (Control (C)) from birth to 14 days of life. Mice were euthanized on day 14, lungs were inflation fixed, and hearts were dissected. (**A**) Right ventricular hypertrophy was measured by Fulton’s index (right ventricle (RV) weight/left ventricle plus septum (LV + S) weight) and compared with age-matched control mice (*n* = 20 per group; * *p* < 0.05 *vs.* SOD2 WT Control mice, # *p* < 0.05 *vs.* SOD2 HET Control mice). Data are shown as mean ± SEM; (**B**) Vascular remodeling was measured by medical wall thickness (MWT). Data are shown as mean ± SEM (*n* = 6–8 vessels per mouse with mice *n* = 6 (SOD2 WT C), 9 (SOD2 HET C), 4 (SOD2 WT CH), and 11 (SOD2 HET CH); * *p* < 0.05 *vs.* SOD2 WT Control mice, # *p* < 0.05 *vs.* SOD2 HET Control mice); and (**C**) Representative HE-stained lung sections (40×) for control (WT and HET) and CH (WT and HET) mice.

**Figure 6:**
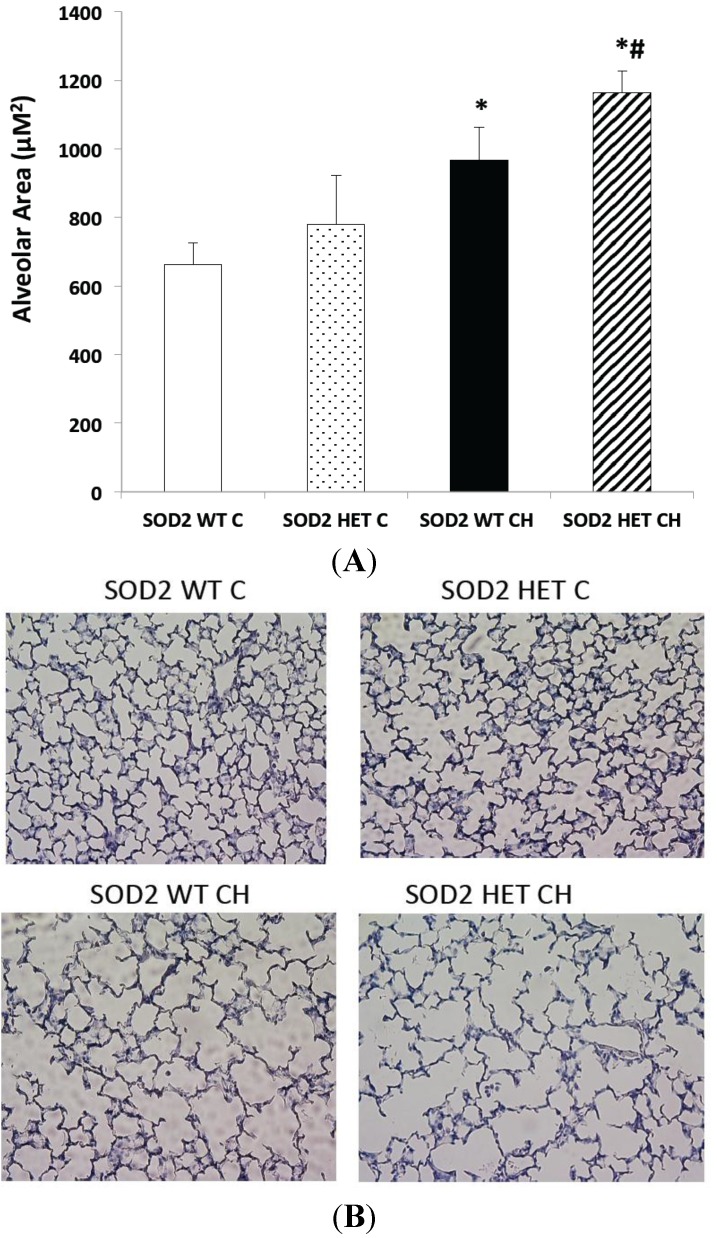
SOD2−/+ mice develop equivalent alveolar simplification in chronic hyperoxia (CH) relative to SOD2+/+ littermates. Neonatal mice (SOD2−/+ (HET) and isogenic SOD2+/+ (WT) littermates) were exposed to chronic hyperoxia (CH) or room air (Control (C)) from birth to 14 days of life. Mice were euthanized on day 14, and lungs were inflation fixed. (**A**) Mean alveolar area was measured on hemotoxylin-stained lung sections using Scion software. Data are shown as mean ± SEM (*n* = 8–10 images per mouse with 4 mice per group; * *p* < 0.05 *vs.* SOD2 WT C mice # *p* < 0.05 *vs.* SOD2 HET Control mice); and (**B**) Representative hematoxylin-stained lung sections (20×) are shown.

Since SOD2 activity was not induced with hyperoxia exposure in either SOD2+/+ or SOD2−/+ PASMC and since SOD2−/+ PASMC had less SOD2 expression and activity at baseline, increased oxidative stress at baseline, and increased PDE5 activity at baseline, we hypothesized that the SOD2−/+ mice would develop more severe lung and pulmonary vascular disease in our hyperoxic mouse model. In both SOD2−/+ and SOD2+/+ littermates, 14 days of CH led to significant RVH as measured by Fulton’s index (*p* < 0.05, Figure 5A) and remodeling in the small pulmonary arteries as measured by medial wall thickness (*p* < 0.05, Figure 5B). Representative vessel images are shown in Figure 5C. However, there was no significant difference between the SOD2−/+ mice and SOD2+/+ littermates in severity of RVH or vascular remodeling. Similarly, both SOD2−/+ and SOD2+/+ mice had significantly increased mean alveolar area relative to SOD2−/+ and SOD2+/+ mice in room air (*p* < 0.05, Figure 6A). However, there was no significant difference between the SOD2−/+ mice and SOD2+/+ littermates in severity of alveolar simplification. Representative hematoxylin images are shown in Figure 6B.

### 2.6. SOD2−/+ Mice Have Increased SOD3 Expression and Unchanged SOD1 Expression in Chronic Hyperoxia (CH)

**Figure 7:**
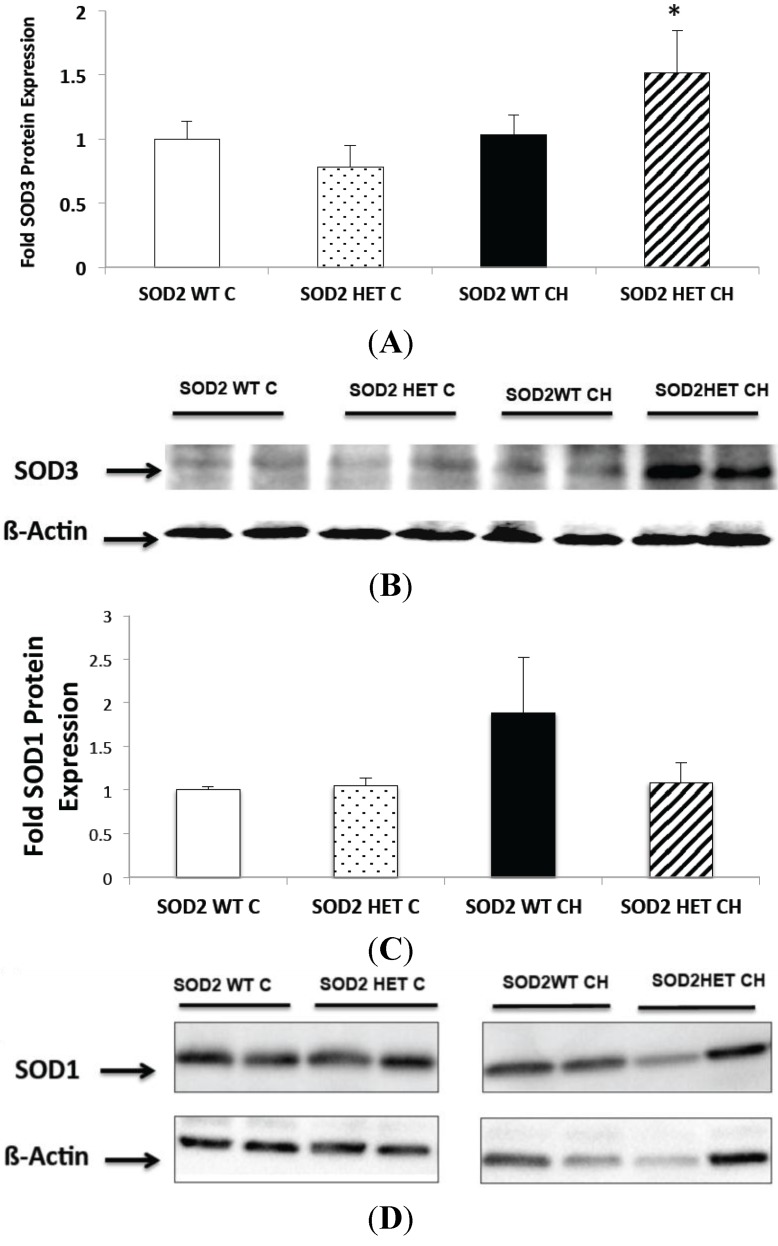
Chronic hyperoxia (CH) exposure increased SOD3 expression in SOD2−/+ mice but does not change SOD1 expression. Neonatal mice (SOD2−/+ (HET) and isogenic SOD2+/+ (WT) littermates) were exposed to chronic hyperoxia (CH) or room air (Control (C)) from birth to 14 days of life. Mice were euthanized on day 14, and lung tissue was harvested. (**A**) Lung SOD3 protein expression was measured by Western blot from WT and HET mice. Data are shown as fold mean ± SEM (*n* = 8; * *p* < 0.05 *vs.* SOD2 HET C); (**B**) Representative Western blots of SOD3 expression is shown with corresponding β-actin; (**C**) Lung SOD1 protein expression was measured by Western blot from WT and HET mice. Data are shown as fold mean ± SEM (*n* = 6); and (**D**) Representative Western blot of SOD1 expression is shown with corresponding β-actin.

Since SOD2−/+ mice did not develop worsened lung and pulmonary vascular disease, we sought to determine if there was compensatory upregulation of other antioxidant enzymes such as SOD1 and SOD3 in lungs from hyperoxia-exposed SOD2−/+ mice. We observed lung SOD3 expression increased significantly in response to CH in the SOD2−/+, but not the SOD2+/+, mice (*p* < 0.05, Figure 7A). Representative Western blots are shown in Figure 7B. On the other hand, when we looked at lung SOD1 expression, there was no significant change in SOD1 expression in response to CH in either SOD2−/+ or SOD2+/+ mice (Figure 7C). Representative Western blots are shown in Figure 7D.

### 2.7. Discussion

In this study, we observed that hyperoxia increases SOD2 expression but not activity in both whole lung and isolated PASMC from mice. Also, we found out that SOD2−/+ mice have approximately 50% of SOD2 protein expression and 70% of SOD2 enzyme activity in their PASMC relative SOD2+/+ PASMC. As a result, SOD2−/+ PASMC have increased mitochondrial ROS at baseline and in hyperoxia. Similarly, we demonstrated that SOD2−/+ PASMC had increased PDE5 activity at baseline relative to SOD2+/+ PASMC. However, in contrast, we observed that heterozygous SOD2−/+ mice can survive normally in room air and with CH exposure for 14 days. SOD2−/+ mice developed RVH, increased MWT, and alveolar simplification in hyperoxia, but it was equivalent to isogenic SOD2+/+ CH mice.

SOD2 plays a critical role in protecting mitochondria from superoxide anions generated during cellular respiration via the electron transport chain. Unlike SOD1 and SOD3, complete inactivation of SOD2 is lethal in mice. SOD2 knockout mice usually die within one to two days [21]*.* Adult human studies have reported a markedly reduced SOD2 protein with decreased enzymatic activity in failing human myocardium [22]. Previous animal studies have shown that SOD2−/+ mice develop myocardial injury-like enhanced fibrosis, hypertrophic cardiomyocytes, and occasional necrosis compared to WT controls [23]. Studies have also shown increased oxidative damage in mitochondria from the livers of SOD2−/+ mice compared with their wild-type littermates [24]. Finally, it has also been published that mitochondria from heterozygous mice showed evidence of increased proton leak, inhibition of respiration, and early and rapid accumulation of mitochondrial oxidative damage [25].

Based on these studies, it seemed reasonable to hypothesize that these mice would be more susceptible to oxidant-mediated injury in a hyperoxia-induced model of lung and pulmonary vascular disease. We studied the impact of SOD2 on hyperoxia-induced lung and vascular injury using SOD2−/+ mice. In contrast to knockout SOD2 (SOD2−/−) mice, SOD2−/+ mice are viable to adulthood with no detectable abnormal phenotype at baseline. In addition, we did not observe any differences in mortality in SOD2−/+ mice exposed to chronic hyperoxia when compared to control SOD2−/+ mice or their wild type littermates. We observed that while PASMC isolated from SOD2−/+ mice have lower baseline SOD2 expression, they demonstrate a greater increase in SOD2 expression following 24 h hyperoxia exposure compared to PASMC from WT mice. While we did not investigate the precise mechanisms of this phenomenon, we speculate that this is likely due to post-translational events such as changes in miRNAs that may regulate the stability and translation of SOD2 transcripts. In addition, we noted that exposure to both short hyperoxia *in vitro* and chronic hyperoxia *in vivo* increased SOD2 expression, but not the SOD2 activity, in isolated PASMC and whole lungs, respectively. We speculate that the disconnect between expression and activity may be due to post-translational modification of the enzyme. Other groups have previously described that SOD2 is nitrated and inactivated in PASMC exposed to hypoxia, which also generates intracellular oxidative stress [17,26]. As we have previously published increased 3-nitrotyrosine staining is present in the CH mice [10], it is reasonable to hypothesize that a similar mechanism is at work here.

Not surprisingly, the PASMC isolated from SOD2−/+ mice had evidence of increased mitochondrial matrix oxidative stress at baseline and in hyperoxia relative to WT PASMC. Our lab has published in the past that exposure to hyperoxia induces mitochondrial matrix oxidative stress, which in turn is associated with increased PDE5 activity in PASMC derived from fetal sheep [8]. Therefore, we hypothesized that the increased mitochondrial matrix oxidative stress observed in the SOD2−/+ PASMC would lead to increased PDE5 activity. In room air, PASMCs from SOD2−/+ mice had significantly increased PDE5 activity compared to SOD2+/+ PASMC. Consistent with our previous results [8], hyperoxia exposure led to increased PDE5 activity in both SOD2−/+ PASMC and SOD2+/+ PASMC. However, there was no difference in the hyperoxia-induced PDE5 activity in SOD2−/+ and SOD2+/+ PASMCs. We speculate that there may be compensatory changes in other proteins that are known to act as intermediaries between oxidative stress and PDE5, such as cGMP-dependent protein kinase [8].

However, since SOD2−/+ PASMC had less SOD2 expression and activity at baseline, increased oxidative stress at baseline, and increased PDE5 activity at baseline, we hypothesized that the SOD2−/+ mice would develop more severe lung and pulmonary vascular disease in our hyperoxic mouse model. Interestingly, we instead found that SOD2−/+ mice developed RVH, pulmonary vascular remodeling, and alveolar simplification equivalent to their isogenic SOD2+/+ littermates. We hypothesize that this interesting finding might be due to compensatory increases in other enzymes responsible for detoxifying superoxide like SOD1 or SOD3. We found there was increased SOD3 protein expression in the SOD2−/+ lungs exposed to CH relative to control SOD2−/+ lungs, but this effect of CH was not observed in SOD2+/+ mice. However, there was no change in SOD1 protein expression in SOD2−/+ and SOD2+/+ mice in chronic hyperoxia. This would suggest that SOD3 and/or other antioxidant systems might be compensating for the decreased SOD2 activity in the SOD2−/+ mice through an as yet unknown mechanism, rendering the SOD2−/+ mice equivalent to the SOD2+/+ mice in terms of disease phenotype. Further studies will be needed to determine the mechanism for this interesting finding.

Our data are consistent with previous hyperoxia studies done in adult SOD2−/+ mice. SOD2−/+ adult mice with levels of lung and heart SOD2 activity approximately 50% of those of SOD2+/+ adult mice developed and survived normally in room air with no demonstrable histologic or ultrastructural abnormalities in the lung or heart. Similarly, they had no difference in their survival curves in 85%–100% O_2_ relative to isogenic SOD2+/+ mice. Interestingly, there were no statistically significant differences between SOD2−/+ and SOD2+/+ mice in the activities of lung SOD1 and GSH peroxidase, but catalase was decreased [27]. Therefore, both groups suggested that in adult mice, 50% of SOD2 activity may be sufficient for normal function in room air and normal response to oxygen toxicity. These findings are consistent with our phenotypic findings in neonatal mice, suggesting that partial SOD2 expression and activity at any age may be sufficient to avoid enhanced hyperoxia-induced cardiovascular injury.

We acknowledge several limitations to our study. Both the length of hyperoxia exposure and the degree of hyperoxia itself were different in the *in vivo vs.* *in vitro* studies. It is therefore possible that the differences in SOD2 responses between neonatal mice and isolated PASMC were a result of the different exposure conditions. Finally, the normoxic conditions used in PASMC experiments (*i.e.*, 21% O_2_) are relatively hyperoxic when compared to the *in vivo* environment, possibly affecting the baseline expression of SOD enzymes.

## 3. Experimental Section

### 3.1. Animal Protocols

This study was approved by the Institutional Animal Care and Use Committee at Northwestern University, Chicago, IL, USA. Newborn C57Bl/6 mouse pups (Charles River, Wilmington, MA, USA) from age-matched litters or isogenic SOD2+/+ and SOD2−/+ mouse pups on a C57Bl/6 background (Jackson Laboratories, Bar Harbor, ME, USA) were exposed to 75% O_2_ in a Plexiglass chamber (Biospherix, Lacona, NY, USA) or to room air (control) within 24 h of birth. Exposure to hyperoxia was continuous for 14 days (chronic hyperoxia or CH), with brief interruptions to rotate the dams every 24 h to prevent oxygen toxicity to the adults. After the completion of the assigned protocol, the pups were euthanized for lung or heart tissue harvest or for formalin fixation and lung inflation as described below. For lung tissue harvest, lungs were perfused with PBS (Mediatech, Manassas, VA, USA) via the right ventricle (RV). Lung tissue was harvested and flash-frozen for subsequent analysis by Western blot or enzyme activity assay as described below. Frozen lung tissue was homogenized into 1× Mg-lysis buffer (Upstate, Charlottesville, VA, USA) supplemented with a protease inhibitor cocktail (Sigma, St. Louis, MO, USA) and a phosphatase inhibitor cocktail (EMD Biosciences, San Diego, CA, USA) as previously described [8].

### 3.2. Isolation of Pulmonary Artery Smooth Muscle Cells (PASMC) and Exposure to Hyperoxia

PASMC were isolated from SOD2−/+ mice and their wild-type littermates (SOD2+/+) at 28 days of life after genotyping. PASMC were isolated as previously described using an iron particle infusion technique [28]. Briefly, a solution of 0.5% (*w*/*v*) agarose + 0.5% iron particles in M199 media (Mediatech) was infused into the PA via the right ventricle (RV). The iron particles are 0.2 μM in diameter and lodge in the small pulmonary arteries (PA). The lungs are inflated with agarose and dissociated. The iron-containing vessels are pulled down with a magnet. After collagenase (80 U/mL) treatment, the vessels are put into a tissue culture dish in M199 media (Mediatech) containing 20% fetal bovine serum (FBS, Hyclone, Logan, UT, USA), and penicillin/streptomycin (Mediatech) to allow cell migration onto the culture dish. The pull down procedure is repeated multiple times to achieve a relatively pure PASMC population and remove any residual iron [28]. SOD2+/+ and SOD2−/+ PASMCs were exposed to 21% O_2_-5% CO_2_ or 95% O_2_-5% CO_2_ in serum free M199 media (Mediatech) containing penicillin/streptomycin (VWR) for 24 h. PASMC were lysed and sonicated in 1× Mg-lysis buffer (Upstate) supplemented with a protease inhibitor cocktail (Sigma-Aldrich, St. Louis, MO, USA) and a phosphatase inhibitor cocktail (EMD Biosciences) as previously described [9].

### 3.3. Western Blot Analysis

Protein concentration of lung or PASMC extracts was measured using the Bradford assay [29]. SOD2 protein expression was assessed via Western blot, which was performed as previously described [30]. Briefly, membranes were blocked at room temperature with 5% nonfat dry milk in Tris-buffered saline containing 0.1% Tween 20 (1× TBST) and incubated overnight at 4 °C with primary antibody in 5% milk + 1× TBST at an appropriate dilution (rabbit anti-SOD2 (1:500; Enzo Life Sciences, Farmingdale, NY, USA); rabbit anti SOD1 (1:500; Enzo Life Sciences); rabbit anti SOD3 (1:500; Enzo Life Sciences); β-actin (1:5000; Sigma-Aldrich) followed by secondary antibody at the appropriate dilution (anti-rabbit (1:750 Cell Signaling, Danvers, MA, USA); anti-mouse (1:2000 dilution, Cell Signaling)). Membranes were then washed and exposed via chemiluminescence (Supersignal West Femto, Thermo Scientific, Hanover Park, IL, USA). Bands were analyzed using a ChemiDoc XRS (BioRad, Hercules, CA, USA) and normalized to β-actin. Data are shown as fold ± SEM relative to control mice.

### 3.4. SOD2 Activity Assay

SOD2 enzyme activity was measured with a commercially available colorimetric assay kit (Cayman Chemical, Ann Arbor, MI, USA) as previously described [31]. Briefly, the assay measures the conversion of tetrazolium salt to formazan dye by superoxide generated by xanthine-xanthine oxidase. Potassium cyanide (1 mM) was added to the lysate (10 μg of total protein) during the assay to inhibit both SOD1 and SOD3. Absorbance was read at 450 nm using a Bio-Rad iMark automated plate reader (BioRad).

### 3.5. Measurement of ROS

PASMC from SOD2−/+ and SOD2+/+ mice were infected with a mitochondrial RoGFP (mitoRoGFP) adenoviral construct. RoGFP is a previously characterized ratiometric fluorescent probe sensitive to cellular oxidative stress. Examination of fluorescence ratios provides a real-time measure of cysteine thiol redox status in the subcellular compartment in which the probe resides. RoGFP–infected SOD2+/+ and SOD2−/+ PASMCs were exposed to 21% O_2_-5% CO_2_ or 95% O_2_-5% CO_2_ for 24 h. After hyperoxia, PASMC aliquots are harvested and analyzed with a DakoCytomation CyAn multilaser flow cytometer using 400 and 485 nm excitation wavelengths and a 535 nm emission wavelength in the NU Flow Cytometry Core Facility [7,9].

### 3.6. PDE5 Activity Assay

PASMC protein was assayed for cGMP-hydrolytic activity using a commercially available kit (Enzo Life Sciences) as previously described [8]. Briefly, the protein was purified over a Centri-Spin 10 column to remove any phosphate contamination (Princeton Separations, Adelphia, NJ, USA). Protein concentration was determined as described above. Each sample was read in four wells—Two without sildenafil and two with sildenafil (100 nM, Sigma-Aldrich), to determine PDE5 specific cGMP-hydrolytic activity. Results were measured using a Bio-Rad iMark automated plate reader (BioRad) at 620 nM. The difference between the pmol cGMP hydrolyzed per mg total protein per minute with and without sildenafil and the pmol cGMP hydrolyzed per mg total protein per minute with sildenafil represents the PDE5-specific cGMP-hydrolytic activity. Results are shown as PDE5-specific pmol cGMP hydrolyzed/min/mg total protein.

### 3.7. Measurement of Right Ventricular Hypertrophy (RVH)

Mouse hearts were dissected, and the right ventricle (RV) and left ventricle plus septum (LV + S) were weighed. Fulton’s Index (RV weight divided by LV + S weight) was used to assess RVH [10].

### 3.8. Measurement of Medial Wall Thickness (MWT)

Pulmonary hypertension was approximated by medial wall thickness (MWT) of small PAs as previously described [10]. Briefly, mouse lungs were inflation fixed at 25 cm H_2_O using 4% formalin, stained with hematoxylin and eosin (H & E), and imaged using an Olympus BX40 microscope (Olympus America, Melville, NY, USA) at 40× with Pixera software (Pixera Corporation, Santa Clara, CA, USA). Eight to ten images per animal were taken and analyzed in a blinded fashion. Medial wall thickness was measured as the ratio of the area of small PA wall over total PA area.

### 3.9. Measurement of Alveolar Area

Alveolar morphometry was performed as previously described [10]. Lung sections were stained with hematoxylin for 16 h, and images were taken on an Olympus BX40 microscope (Olympus America) at 20× with Pixera software (Pixera Corporation). Eight to ten images per animal were taken and analyzed in a blinded fashion. Mean alveolar area was measured using Scion software (Informer Technologies, Shingles Springs, CA, USA).

### 3.10. Statistical Analysis

All data are expressed as means ± SEM, with “*n*” representing the number of animals in each group and with significance at *p* < 0.05. Results were analyzed by *t*-test or ANOVA with *post-hoc* Bonferroni’s analysis as appropriate using Prism software (Graphpad Software Inc., San Diego, CA, USA).

## 4. Conclusions

In conclusion, our study suggests that neonatal SOD2−/+ mice are no more susceptible to hyperoxic lung and cardiovascular injury than wild-type littermates. While 50% of SOD2 expression may be sufficient in the neonatal period for normal development in room air, these mice are still susceptible to hyperoxia-induced lung and vascular injury that is equivalent to wild-type mice. Thus, while loss of SOD2 does not appear to be pathogenic, we speculate that overexpression of SOD2 in the neonatal period might be protective in hyperoxia-induced lung and cardiovascular injury, similar to the beneficial effect it has in adult lung injury [15]. Further studies will be needed to determine both the mechanism of compensation in the SOD2−/+ mice as well as SOD2 overexpression in hyperoxia-induced pulmonary and cardiovascular injury.
